# Novel Cell Wall Antifungals Reveal a Special Synergistic Activity in *pbr1* Mutants Resistant to the Glucan Synthesis Antifungals Papulacandins and Echinocandins

**DOI:** 10.3389/fmicb.2019.01692

**Published:** 2019-07-24

**Authors:** Rodrigo Berzaghi, Attila Agócs, María A. Curto, Gergely Gulyás-Fekete, Béla Kocsis, Juan C. Ribas, Tamás Lóránd

**Affiliations:** ^1^Instituto de Biología Funcional y Genómica, Consejo Superior de Investigaciones Científicas, Universidad de Salamanca, Salamanca, Spain; ^2^Department of Biochemistry and Medical Chemistry, University of Pécs Medical School, Pécs, Hungary; ^3^Department of Medical Microbiology and Immunology, University of Pécs Medical School, Pécs, Hungary

**Keywords:** antifungals, inhibitors, glucan synthase, echinocandin, cell wall

## Abstract

A series of 4*-*(arylmethylene)-3-isochromanones have been prepared with base-catalyzed Knoevenagel condensation starting from 3-isochromanone and aromatic aldehydes. The outcome of the reaction- the isomeric composition of the products depends on the aromatic aldehyde applied. These reactions afforded mostly the more stable *E*-diastereoisomer, but some condensations resulted in the Z-diastereoisomer or mixture of the stereoisomers (**1**–**16**). The products showed antifungal effect against some pathogenic fungi. We wanted to extend this study and to synthesize a new generation of 4*-*(arylmethylene)-3-isochromanones. These condensations led mostly to *E*-diastereoisomers (**17**–**30**). The structure verifications were performed by FT IR, ^1^H and^13^C NMR methods. Both the **1**–**16** and the novel **17**–**30** compounds have been screened against the three yeast models, fission yeast *Schizosaccharomyces pombe* (wild-type, and *pbr1-6* and *pbr1-8* mutants resistant to specific cell wall synthesis inhibitors), budding yeast *Saccharomyces cerevisiae* (wild-type and *pbr1-1*) and pathogenic yeast *Candida albicans* (wild-type, ATCC 26555, 90028 and SC5314). Osmotic protection with sorbitol attenuated the *in vivo* inhibition in living cells suggesting a cell wall-specific antifungal effect. Moreover, the *S. pombe* wild-type and mutant strains were tested for their resistant or sensitive *in vitro* β(1,3)-glucan synthase (GS) activity. We found both *in vivo* in living cells and *in vitro* in the enzymatic GS assay a synergistic effect of higher sensitivity of the *pbr1* mutants resistant to the specific GS inhibitors papulacandins and echinocandins. These results may provide new insights into new strategies of combined antifungal therapy of GS inhibitors directed against spontaneous mutants resistant to echinocandins.

## Introduction

**FIGURE 1:**
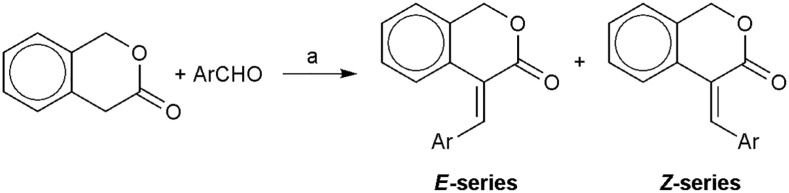
Synthetic route of the 4-(arylmethylene)-3-isochromanones; **Reagents and conditions:** a) piperidine, 140°C, Ar.

**FIGURE 2:**
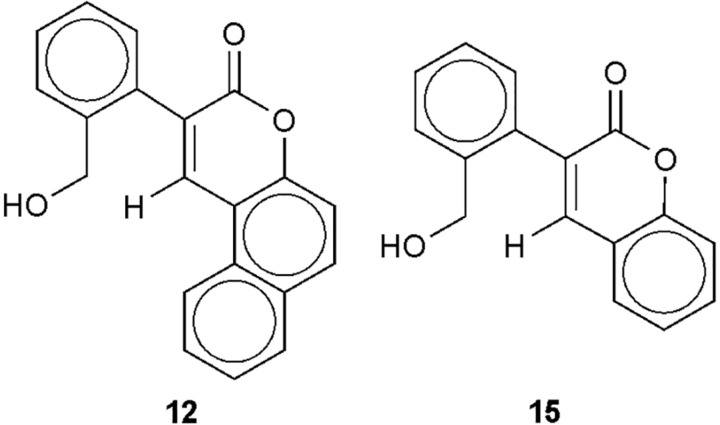
The structure of coumarines (**12,15**) formed in the condensations with some hydroxyaldehydes.

In our previous studies we have dealt with the synthesis of some E-2-arylmethylene-1-tetralones and E-2-heteroarylmethylene-1-tetralones applying base-cataly**z**ed aldol condensation. These compounds were examined for their antimycotic activity using some clinical isolates of the pathogenic fungi *Candida albicans*, *Cryptococcus neoformans*, etc. Some of them showed a high antifungal activity with low MIC (Minimal Inhibitory Concentration) values of 1.5 μg/mL and several of the tested compounds exerted better activity than the commercial antifungal standards (Al-Nakib et al., 2001). With the aim of finding new and better specific antifungal inhibitors, we wished to expand these investigations to the analogous α,β-unsaturated heterocyclic ketones. Therefore the principle of the natural product based synthesis was utilized and a molecular library of 4-(arylmethylene)-3-isochromanones was prepared via base-cataly**z**ed Knoevenagel condensations, i.e., the first generation of isochromanones (**1—16**, see in Figure 1 and Table 1) (Lóránd et al., 2002). Using phenolic aldehydes –with ortho-OH group- in this reaction resulted in the formation of the corresponding coumarines **(12, 15)** due to a second intramolecular reaction (Figure 2) (Lóránd et al., 2002). (Compounds of isochromane skeleton occur in the nature as the tricyclic fusarubin, showing antibacterial and antifungal effect) (Ruelius and Gauhe, 1950). Several members of the first generation of 4-(arylmethylene)-3-isochromanones showed excellent antifungal activity using some clinical isolates of *C. albicans*, *C. neoformans*, etc. (Lóránd et al., 1998). In order to collect a larger molecular library for biological investigations we planned to prepare a new class of isochromanones. We wished to explore the antifungal activity of the two families of isochromanones. The *in vivo* inhibitory capacity of the first family against clinical isolates of *C. albicans* or *C. neoformans* has been shown (Lóránd et al., 1998). Thus our aim was to extend or investigate the *in vivo* inhibitory capacity on the living cells of both families of isochromanones and to study the possible *in vitro* inhibitory capacity of both families of the β(1,3)-glucan synthase (GS) activity in order to find new families of specific fungal cell wall synthesis inhibitors. The cell wall is an essential structure for the fungal cells that is absent in animal cells (Free, 2013; Cortés et al., 2016; Gow et al., 2017; Hopke et al., 2018) therefore representing a very useful target in the search for new, more efficient and selective drugs for the treatment of invasive fungal infections without causing toxicity in the animal host cells (Bal, 2010; Hector and Bierer, 2011; Brown et al., 2012; Kathiravan et al., 2012; Cortés et al., 2019). In this study, we used the following wild-type or papulacandin and echinocandin resistant mutant strains: *Schizosaccharomyces pombe* wild-type, and pbr1-8 and pbr1-6 resistant mutants (Castro et al., 1995; Martins et al., 2011), *Saccharomyces cerevisiae* wild-type and pbr1-1 resistant mutant (Castro et al., 1995), and several *C. albicans* wild-type strains.

**TABLE 1:** Structure and isomeric composition of the first generation of second generation of isochromanones (**1–16**).

**Compound**	**Ar**	**Isomeric composition**	
		**% (*E*)**	**% (*Z*)**
**1*E*, *Z***	Ph	60	40
**2*E***	4′-COOH-C_6_H_4_	100	–
**3*E***	3′-pyridyl	100	–
**4*E*, *Z***	2′-(1-methyl)-pyrrolyl	66	33
**5*E***	2′-pyrrolyl	–	100
**6*E***	2′,6′-Cl_2_-C_6_H_3_	–	100
**7*E***	2′-OCH_3_-C_6_H_4_	100	–
**8*E*, *Z***	2′-Cl-C_6_H_4_	70	30
**9*E***	2′,3′-(OCH_3_)_2_-C_6_H_3_	100	–
**10*E***	2′,4′-(OCH_3_)_2_-C_6_H_3_	100	–
**11*E*, *Z***	2′-NO_2_-C_6_H_4_	33	66
**12**	2′-OH-1′-naphthyl	coumarine	
**13*E*, *Z***	2′-furyl	66	33
**14*E***	2′-Br-C_6_H_4_	100	–
**15**	2′-OH-C_6_H_4_	coumarine	
**16*E***	3′-OH-C_6_H_4_	100	–

## Materials and Methods

### General Procedures for the Synthesis of Compounds

All of the reagents were purchased from Sigma-Aldrich Company and were used without further purifications. The purification of the novel compounds was performed with column chromatography on Sigma-Aldrich silica gel (pore size: 60 Å, particle size 230–400 mesh). The analytical thin-layer chromatography was performed on Merck silica gel plates (60 F_254_) and the eluents used are described in the next chapter. NMR spectra were recorded on a Bruker Avance III Ascend 500 spectrometer (500/125 MHz for ^1^H/^13^C); chemical shifts are referenced to residual solvent signals. Measurements were performed at a probe temperature of 298 K in solution with an appropriate solvent. The FT IR spectra were run on an Impact 400 (Nicolet) FT IR spectrophotometer in KBr pellets using a KBr pellet as the background reference spectrum. Infrared spectra were obtained between 400 and 4000 cm^–1^ with a spectral resolution of 4 cm^–1^ Melting points were measured with a Boethius hot plate apparatus and are uncorrected. The spectroscopic data of the title compounds can be found in the Supplementary Material.

#### Synthesis of 4-Arylmethylene-3-Isochromanones **(17–30)** (Lóránd et al., 2002)

The equimolar mixture of 3-isochromanone (3.38 mmol) and the corresponding aromatic aldehyde (3.38 mmol) was heated (140°C) in the presence of catalytic amount of piperidine (four drops) in argon atmosphere under stirring for 1 h. Then, the reaction mixture was taken up in ethanol and cooled down. The crystals separated were filtered off and washed with cold ethanol. The products were purified by means of column chromatography. (See details at the compounds.) The conversion was always 100%.

#### Synthesis of *E*-4-[(4′-Nitrophenyl)Methylene]-3- Isochromanone **(17*E*)** and *Z*-4-[4′-Nitrophenyl)- Methylene]-3-Isochromanone **(17*Z*)**

Obtained from a mixture of 3-isochromanone (0.50 g; 3.38 mmol) and 4-nitrobenzaldehyde (0.51 g, 3.38 mmol) with the above method. The reaction mixture was separated by column chromatography (silica gel, CH_2_Cl_2_/CH_3_OH = 10:0.01) to afford **17*E*** (85%) and **17*Z*** (15%) based on separation (0.69 g, 72%). **17*E*** *R*_f_ = 0.37 (silica gel, dichloromethane/methanol = 10:0.05), yellow crystalline solid from methanol. m.p. 145–146°C. IR ν_max_ (cm^–1^) (KBr) 1726 (st, C = O). ^1^H NMR (500 MHz, dmso-d_6_) δ (ppm) 5.47 (s, 2H), 7.13 (d, *J* = 7.6 Hz, 1H), 7.20 (t, *J* = 7.2 Hz, 1H), 7.38 (dt, *J* = 7.6, 0.7 Hz, 1H), 7.47 (d, *J* = 7.6 Hz, 1H), 7.72 (d, *J* = 8.7 Hz, 2H), 7.79 (s, 1H), 8.21 (d, *J* = 8.7 Hz, 2H).^13^C NMR. (125 MHz, dmso-d_6_) δ (ppm) 68.8, 123.7, 125.8, 127.0, 128.1, 128.2, 129.0, 129.1, 130.4, 133.5, 135.0, 141.3, 147.2, 167.1, *^3^J*(Hα-C3) = 7.1 Hz. Anal Calcd for C_16_H_11_NO_4_: C, 68.33; H, 3.94; Found: C, 68.22; H, 4.11.

##### *Z*-4-[(4′-nitrophenyl)methylene]-3-isochromanone **(17*Z*)**

It is a yellow crystalline solid from methanol. m.p. 202°C (dec.). IR ν_max_ (cm^–1^) (KBr) 1723 (st, C = O). Anal Calcd for C_16_H_11_N_1_O_4_: C, 68.33, H, 3.94; Found: C, 68.12; H, 4.20.

#### Synthesis of *E*- 4-[(2′,3′,4′-Trimethylphenyl)Methylene]-3-Isochromanone **(18*E*)**

Prepared from a mixture of 3-isochromanone (0.50 g; 3.38 mmol) and mesitaldehyde (98%) (0.51 g, 3.37 mmol) with the above method (0.82 g, 89%). *R*_f_ = 0.52 (silica gel, dichloromethane/methanol = 10:0.01), m.p.155°C (dec.) yellow crystalline solid from methanol. IR ν_max_ (cm^–1^) (KBr) 1726 (st, C = O). Anal Calcd for C_19_H_18_O_2_: C, 81.99; H, 6.52; Found: C, 82.18; H, 6.65.

#### Synthesis of *E*-4-[(4′-Methylphenyl)Methylene]- 3-Isochromanone **(19*E*)**

Prepared from a mixture of 3-isochromanone (0.50 g; 3.38 mmol) and *p*-tolualdehyde (0.41 g, 3.38 mmol) with the above method. The reaction mixture was purified via column chromatography (silica gel, toluene/ethyl acetate = 95.5:4.5) to afford **19*E*** (0.49 g, 58%). *R*_f_ = 0.49 (silica gel, toluene/ethyl acetate = 10:1), m.p.125–26°C, yellow crystalline solid from methanol. IR ν_max_ (cm^–1^) (KBr) 1716 (st, C = O). Anal Calcd for C_17_H_14_O_2_: C, 81.58; H, 5.64; Found: C, 80.69; H, 5.70.

#### Synthesis of *E*-4-[(2′-Methylphenyl)Methylene]- 3-Isochromanone **(20*E*)**

Prepared from a mixture of 3-isochromanone (0.50 g; 3.38 mmol) and *o*-tolualdehyde (0.41 g, 3.38 mmol) with the above method. The reaction mixture was purified via column chromatography (silica gel, toluene/ethyl acetate = 95.5:4.5) to afford **20*E*** (0.42 g, 50%). *R*_f_ = 0.49 (silica gel, toluene/ethyl acetate = 10:1), m.p.78°C (dec.), yellow crystalline solid from methanol. IR ν_max_ (cm^–1^) (KBr) 1722 (st, C = O). Anal Calcd for C_17_H_14_O_2_: C, 81.58; H, 5.64; Found: C, 81.70; H, 5.79.

#### Synthesis of *E*-4-[(4′-Methoxyphenyl)Methylene]-3-Isochromanone **(21*E*)**

Prepared from a mixture of 3-isochromanone (0.50 g; 3.38 mmol) and 4-anisaldehyde (0.47 g, 3.34 mmol) with the above method. The reaction mixture was purified via column chromatography (silica gel, toluene/ethyl acetate = 95.5:4.5) to afford **21*E*** (0.73 g, 81%). *R*_f_ = 0.43 (silica gel, toluene/ethyl acetate = 10:1), m.p.129–131°C, yellow crystalline solid from methanol. IR ν_max_ (cm^–1^) (KBr) 1707 (st, C = O). Anal Calcd for C_17_H_14_O_3_: C, 76.68; H, 5.30; Found: C, 76.79; H, 5.49.

#### Synthesis of *E*-4-[(3′-Methoxyphenyl)Methylene]-3-Isochromanone **(22*E*)**

Prepared from a mixture of 3-isochromanone (0.50 g; 3.38 mmol) and 3-anisaldehyde (0.47 g, 3.34 mmol) with the above method. The reaction mixture was purified via column chromatography (silica gel, toluene/ethyl acetate = 95.5:4.5) to afford **22*E*** (0.59g, 66%), *R*_f_ = 0.43 (silica gel, toluene/ethyl acetate = 10:1), m.p.102–103°C, yellow crystalline solid from methanol. IR ν_max_ (cm^–1^) (KBr) 1719 (st, C = O). Anal Calcd for C_17_H_14_O_3_: C, 76.68; H, 5.30; Found: C, 76.79; H, 5.48.

#### Synthesis of *E*-4-[(4′-Chlorophenyl)Methylene]- 3-Isochromanone **(23*E*)**

Prepared from a mixture of 3-isochromanone (0.50 g; 3.38 mmol) and 4-chlorobenzaldehyde (0.48 g, 3.34 mmol) with the above method. The reaction mixture was purified via column chromatography (silica gel, toluene/ethyl acetate = 95.5:4.5) to afford **23*E*** (0.68 g, 74%), *R*_f_ = 0.44 (silica gel, toluene/ethyl acetate = 10:1), m.p.163–165°C, yellow crystalline solid from methanol. IR ν_max_ (cm^–1^) (KBr) 1716 (st, C = O). Anal Calcd for C_16_H_11_ClO_2_: C, 70.99; H, 4.10; Found: C, 71.15; H, 4.22.

#### Synthesis of *E*-4-[(3′-Nitrophenyl)Methylene]- 3-Isochromanone **(24*E*)** and *Z*-4-[(3′-Nitrophenyl) -Methylene]-3-Isochromanone **(24*Z*)**

Obtained from a mixture of 3-isochromanone (0.50 g; 3.38 mmol) and 3-nitrobenzaldehyde (0.51 g, 3.38 mmol) with the above method. The reaction mixture was separated by column chromatography (silica gel, dichloromethane/ methanol = 10:0.05) to afford 65% **24*E*** and 35% **24*Z*** stereoisomers (0.56 g, 58%). **24*E***: *R*_f_ = 0.55 (silica gel, dichloromethane/methanol = 10:0.1), m.p. 183°C, yellow crystalline solid from methanol. IR ν_max_ (cm^–1^) (KBr) 1720 (st, C = O). Anal Calcd for C_16_H_11_N_1_O_4_: C, 68.33; H, 3.94; Found: C, 68.22; H, 4.19.

##### *Z*-4-[(3′-nitrophenyl)methylene]-3-isochromanone **(24Z)**

*R*_f_ = 0.65 (silica gel, dichloromethane/methanol = 10:0.1), Yellow crystals from methanol, m.p.178–179°C, IR ν_max_ (cm^–1^) (KBr) 1724 (st, C = O), Anal Calcd for C_16_H_11_NO_4_: C, 68.33; H, 3.94; Found: C, 68.47; H, 4.17.

#### Synthesis of *E*-4-[(3′,4′,5′-Trimethoxyphenyl)Methylene]-3-Isochromanone **(25*E*)**

Prepared from a mixture of 3-isochromanone (0.50 g; 3.38 mmol) and 3,4,5-trimethoxybenzaldehyde (95%) (0.70 g, 3.38 mmol) with the above method. The reaction mixture was purified via column chromatography (silica gel, toluene/ethyl acetate = 95.5:4.5) to give **25*E*** (0.75 g, 68%), *R*_f_ = 0.34 (silica gel, toluene/ethyl acetate = 8:2), m.p.165–167°C, yellow crystalline solid from methanol. IR ν_max_ (cm^–1^) (KBr) 1723 (st, C = O). Anal Calcd for C_19_H_18_O_5_: C, 69.93; H, 5.56; Found: C, 70.11; H, 5.64.

#### Synthesis of *E*-4-[(3′,4′-Dimethoxyphenyl)Methylene]-3-Isochromanone **(26*E*)**

Prepared from a mixture of 3-isochromanone (0.50 g; 3.38 mmol) and veratraldehyde (95%) (0.60 g, 3.43 mmol) with the above method. The reaction mixture was purified via column chromatography (silica gel, toluene/ethyl acetate = 95.5:4.5) to give **26*E*** as a faint yellow crystalline solid from methanol (0.71g, 64%), *R*_f_ = 0.36 (silica gel, toluene/ethyl acetate = 8:2), m.p.129–131°C. IR ν_max_ (cm^–1^) (KBr) 1717 (st, C = O). Anal Calcd for C_18_H_16_O_4_: C, 72.96; H, 5.44; Found: C, 73.09; H, 5.58.

#### Synthesis of *E*-4-[(3′,4′-Methylenedioxyphenyl)Methylene]-3-Isochromanone **(27*E*)**

Obtained from a mixture of 3-isochromanone (0.50 g; 3.38 mmol) and piperonal (0.51 g, 3.37 mmol) with the method above. The reaction mixture was separated by column chromatography (silica gel, toluene/ethyl acetate = 95.5:4.5) to afford 40% **27*E*** and 60 % **27*Z*** stereoisomers (0.63 g, 66%). **27*E*** *R*_f_ = 0.42 (silica gel, toluene/ethyl acetate = 10:1), m.p.153–155°C, yellow crystalline solid from methanol. IR ν_max_ (cm^–1^) (KBr) 1716 (st, C = O). Anal Calcd for C_17_H_12_O_4_: C, 72.85; H, 4.32; Found: C, 72.95; H, 4.50.

##### *Z*-4-[(3′,4′-methylenedioxyphenyl)methylene]-3-isochromanone **(27*Z*)**

*R*_f_ = 0.43 (silica gel, toluene/ethyl acetate = 10:1), m.p.157–158°C, yellow crystalline solid from methanol. IR ν_max_ (cm^–1^) (KBr) 1715 (st, C = O). Anal Calcd for C_17_H_12_O_4_: C, 72.85; H, 4.32; Found: C, 72.99; H, 4.48.

#### Synthesis of *E*-4-[(3′- Chlorophenyl)Methylene]-3-Isochromanone **(28*E*)**

Obtained from a mixture of 3-isochromanone (0.50 g; 3.38 mmol) and 3-chlorobenzaldehyde (95%) (0.50 g, 3.38 mmol) with the above method. The reaction mixture was separated by column chromatography (silica gel, dichloromethane) to afford 80% **28*E*** and 20% **28*Z*** (0.61 g, 67%). **28*E*** *R*_f_ = 0.37 (silica gel, dichloromethane), m.p.132–133°C, yellow crystalline solid from methanol. IR ν_max_ (cm^–1^) (KBr) 1724 (st, C = O). Anal Calcd for C_16_H_11_ClO_2_: C, 70.99; H, 4.10; Found: C, 71.18; H, 4.23.

##### *Z*-4-[(3′-chlorophenyl)methylene]-3-isochromanone **(28Z)**

*R*_f_ = 0.49 (silica gel, dichloromethane), m.p 116–117°C, yellow crystalline solid from methanol. IR ν_max_ (cm^–1^) (KBr) 1721 (st, C = O). Anal Calcd for C_16_H_11_ClO_2_: C, 70.99; H, 4.10; Found: C, 71.22; H, 4.31.

#### Synthesis of *E*-4-[(4′- Hydroxyphenyl)Methylene]-3-Isochromanone **(29*E*)**

Prepared from a mixture of 3-isochromanone (0.50 g; 3.38 mmol) and 4-hydroxybenzaldehyde (0.41 g, 3.38 mmol) with the above method. The reaction mixture was purified via column chromatography (silica gel, dichloromethane/methanol = 10:0.05) to give **29*E*** as a faint yellow crystalline solid from methanol (0.62 g, 73%). *R*_f_ = 0.14 (silica gel, dichloromethane/methanol = 10:0.15), m.p. 212–213°C. IR ν_max_ (cm^–1^) (KBr) 3444 (st, OH), 1703 (st, C = O). Anal Calcd for C_16_H_12_O_3_: C, 76.18; H, 4.79; Found: C, 76.30; H, 4.91.

#### Synthesis of *E*- 4-[(4′-Hydroxy-3′-Methoxyphenyl)Methylene]-3-Isochromanone **(30*E*)**

Prepared from a mixture of 3-isochromanone (0.50 g; 3.38 mmol) and vanillin (0.51 g, 3.38 mmol) with the above method. The reaction mixture was purified via column chromatography (silica gel, dichloromethane/methanol = 10:0.05) to give **30*E*** as a faint yellow crystalline solid from methanol (0.60 g, 63%), *R*_f_ = 0.24 (silica gel, dichloromethane/methanol = 10:0.1), m.p. 140–141°C. IR ν_max_ (cm^–1^) (KBr) 3346 (st, OH), 1696 (st C = O). Anal Calcd for C_17_H_14_O_4_: C, 72.33; H, 5.00; Found: C, 72.41; H, 5.12.

### Biological Assays

#### Antifungal Assay

Strains used in these experiments: *C. albicans* wild-type ATCC 26555, *C. albicans* wild-type SC5314 (ATCC MYA-2876), *C. albicans* wild-type ATCC 90028, *S. cerevisiae* wild-type CVX12-3A (*MATα ura3-373-251-328 his4-34 leu2-3-112*), *S. cerevisiae MATα pbr1-1 leu1 ura3-373-251-328, S. pombe* wild-type 972 h^–^, *S. pombe pbr1-8 leu1-32* h^–^*, S. pombe pbr1-6 leu1-32* h^–^.

The antifungal activity of isochromanones was determined with agar dilution method by using Sabouraud-chloramphenicol agar as previously published (Zacchino et al., 1997, 1999). Stock solutions of tested compounds (10 mg/mL in DMSO) were diluted to afford serial 2-fold dilutions that were added to each medium yielding in concentrations ranging from 0.10 to 250 μg/ mL. The final concentration of DMSO in the assay was not higher than 2.5%.

As standard amphotericin B was applied (Inj. FUNGIZONE 50mg Bristol-Myers Squibb Hungary Kft.). The antifungal caspofungin was a generous gift from Merck Sharp & Dohme.

#### Determination of MIC (Minimum Inhibitory Concentration) Value by Macro and Micro Tube Dilution Method (Alfa et al., 1993; Washington et al., 2006; Espinel-Ingroff and Pfaller, 2007)

Standard YEPD (Yeast Extract, Peptone, Dextrose) (Sherman, 1990), YEPD+S (YEPD with 1.2M sorbitol), YES (Yeast Extract, Dextrose and Supplements of Adenine, Histidine, Leucine, Uracil and Lysine) (Alfa et al., 1993) and YES+S (YES with 1.2 M sorbitol) (Ribas et al., 1991; Ishiguro et al., 1997), media were used for determination of MIC values of the tested compounds. A stock solution of 10 mg/ml of each tested compounds was prepared in DMSO and serial dilutions in DMSO were prepared. The final concentration of DMSO was 2% in the YEPD medium. This concentration of DMSO does not influence the viability and sensitivity of the tested yeast strains. The serial dilutions of compounds were dispensed into test tubes with 1 ml of YEPD or YEPD+S to make final concentrations of 200, 100, 50, 25, 12.5, 6.26, 3.125, 1.6, 0.8 and 0.4 μg/ml. The tested yeast strains were previously grown in YEPD, YEPD+S, YES or YES+S agar plates at 30°C for 48 h.

For inoculation of YEPD or YEPD+S macro test tubes a suspension of tested fungus was produced with 10^5^ cfu/ml (cfu - colony forming unit). 10 μl of suspension was used for inoculation of each tube. After 48 h incubation at 30°C the tubes were checked. The MIC value is the lowest concentration of the tested compound that could inhibit the fungal growth and the medium remained clear. From each tube 10 μl culture was plated on YEPD agar medium to check the contamination. The MIC tests were carried out in three parallels.

Micro-culture assays of large numbers of samples were analyzed as described) (Martins et al., 2011). Essentially early log-phase cells were grown in YEPD, YEPD+S, YES or YES+S (1.2 M sorbitol) medium at 30°C, and diluted to cell density of 4 X 10^6^ cells/ml in YEPD, YEPD+S, YES or YES+S medium and aliquots of 100 μl were dispensed into each small test tube (3 ml test tube size). Then, another 100 μl of YEPD, YEPD+S, YES or YES+S with the corresponding dilution of each compound at 2X concentration (4% DMSO) was added. The final volume was 200 μl, with the cells diluted to of 2 X 10^6^ cells/ml and each compound diluted to 1X final concentration, containing increasing concentrations of antifungal (0, 1, 2, 5, 10, 20, 50, 100 and 200 μg/ml) or an equivalent volume of solvent (2% DMSO). The cells were grown at 30°C in an orbital roller and turbidity was analyzed after 24 and 48 h of incubation. The MIC was determined as the minimal concentration of tested compound that produced complete cell growth inhibition and kept the medium clear. The MIC values were calculated from at least three independent experiments.

According to the previous experiments of the authors the GS-specific antifungals showed the following *in vivo* MIC values – in brackets – for the different *S. pombe* strains given in μg/ml: *S. pombe* wild-type: papulacandin (5), enfumafungin (10), pneumocandin (5), caspofungin (10); *S. pombe pbr1-8* mutant: papulacandin (>100), enfumafungin (>100), pneumocandin (>100), caspofungin (50); *S. pombe pbr1-6* mutant: papulacandin (>100), enfumafungin (>100), pneumocandin (50), caspofungin (30) (Martins et al., 2011). In this study we have used caspofungin as a standard control of the *in vivo* MIC values in *S. pombe, S. cerevisiae and C. albicans* cells.

#### Enzyme Preparation and β(1,3)-Glucan Synthase Assay

β(1,3)-D-glucan synthase assay was executed as published previously) (Ishiguro et al., 1997; Martins et al., 2011). Cell extracts were obtained from early log-phase cells grown in YES medium at 28°C. Membrane enzyme extracts were resuspended in buffer A (50 mM Tris-HCl, pH 7.5, 1 mM EDTA, and 1 mM β-mercaptoethanol) containing 33% glycerol and 50 μM GTPγS and stored at −80°C. The standard assay mixture contained 5 μl of enzyme (15–25 μg protein), 150 μM GTPγS, and 2 μl of increasing concentrations of tested compound (0, 0.01, 0.02, 0.05, 0.1, 0.2, 0.5, 1, 2, 5, 10 and 20 μg in 2 μl of DMSO, from dilutions of a stock solution of 10 mg/ml in DMSO and kept at −20°C) or an equivalent volume of 2 μl of solvent DMSO, in a total volume of 40 μl. The reaction was incubated for 30 min at 30°C and stopped by addition of 1 ml 10% trichloroacetic acid. The IC50 was determined as the concentration of tested compound that produced half-maximal inhibitory concentration of the *in vitro* GS activity and the IC30 was the concentration of tested compound that produced 30% inhibition of the maximal activity. All reactions were carried out in duplicate, and the values were calculated from three independent cell cultures.

According to the previous experiments of the authors the control antifungal caspofungin used in this study showed the following *in vitro* IC50 values – in brackets - of the enzymatic GS activity for the different *S. pombe* strains given in μg/ml: *S. pombe* wild-type: caspofungin (0.3); *S. pombe pbr1-8* mutant: caspofungin (250); *S. pombe pbr1-6* mutant: caspofungin (150) (Martins et al., 2011). In this study we have used caspofungin as a standard control of the IC50 and IC30 values for *in vitro* assays of enzymatic GS activity.

## Results and Discussion

### Synthesis and Structure Elucidation

The selection of the aromatic aldehydes was based on the existing structure-antimicrobial activity relationships. In addition, we wanted to study the effect of the aromatic substituent on the stereo-composition of the reaction mixture. The synthetic route was a solvent free one step method starting from 3-isochromanone and the corresponding aromatic aldehyde using catalytic amount of piperidine (Lóránd et al., 2002). (Under more basic conditions the 3-isochromanone ring undergoes hydrolysis) (Barbier, 1987). To avoid the air oxidation the condensations were carried out under argon atmosphere at 140°C. With this procedure the degradation of the starting 3-isochromanone can be avoided and good yields (50–80%) can be achieved. By this route we have prepared the second generation of isochromanones (**17–30**, see in Table 2 and Figure 1) with different substitution patterns- with both electron-withdrawing and electron-donating groups in the aromatic nucleus.

**TABLE 2:** Structure and isomeric composition of the second generation of isochromanones (**17**–**30**).

**Compound**	**Ar**	**Isomeric composition**	
		**% (*E*)**	**% (*Z*)**
**17*E*, *Z***	4′-NO_2_-C_6_H_4_	85	15
**18*E***	2′,4′,6′-(CH_3_)_3_-C_6_H_2_	100	–
**19*E***	4′-CH_3_-C_6_H_4_	100	–
**20*E***	2′-CH_3_-C_6_H_4_	100	–
**21*E***	4′-OCH_3_-C_6_H_4_	100	–
**22*E***	3′-OCH_3_-C_6_H_4_	100	–
**23*E***	4′-Cl-C_6_H_4_	100	–
**24*E*, *Z***	3′-NO_2_-C_6_H_4_	65	35
**25*E***	3′,4′,5′-(OCH_3_)_3_-C_6_H_2_	100	–
**26*E***	3′,4′-(OCH_3_)_2_-C_6_H_3_	100	–
**27*E*, *Z***	3′,4′-(OCH_2_O)-C_6_H_3_	40	60
**28*E*, *Z***	3′-Cl-C_6_H_4_	80	20
**29*E***	4′-OH-C_6_H_4_	100	–
**30*E***	3′-OCH_3_-4′-OH-C_6_H_4_	100	–

The degree of conversion was in each case 100%. These condensations generally yield from the two possible stereoisomers solely the *E*-stereoisomer. However, in the case of sterically hindered educts the Z-stereoisomer forms (Dimmock and Wong, 1976; Rossi et al., 2010). In four cases a mixture of *E/Z*-isomers was obtained (**17**, **24**, **27** and **28**). Regarding the influence of the aromatic substituents, compounds **17**, **24** and **28** contain electron-withdrawing substituents in the aromatic ring.

The structure of the title compounds was supported by the FT IR spectra, too. The strongest band of the FT IR spectra belongs to the CO stretching vibrations lying mostly in the region of 1715–1726 cm^–1^ with the exception of compounds **21**, **29–30**. Similar absorption maxima of the CO stretching were observed at analogous compounds – **11*E* –*Z*** (Keresztury et al., 2004). The decreased νCO frequency of the two hydroxyl compounds (**29–30**) can be explained with the intermolecular hydrogen bond decreasing the absorption maxima (Silverstein et al., 2015).

**FIGURE 3:**
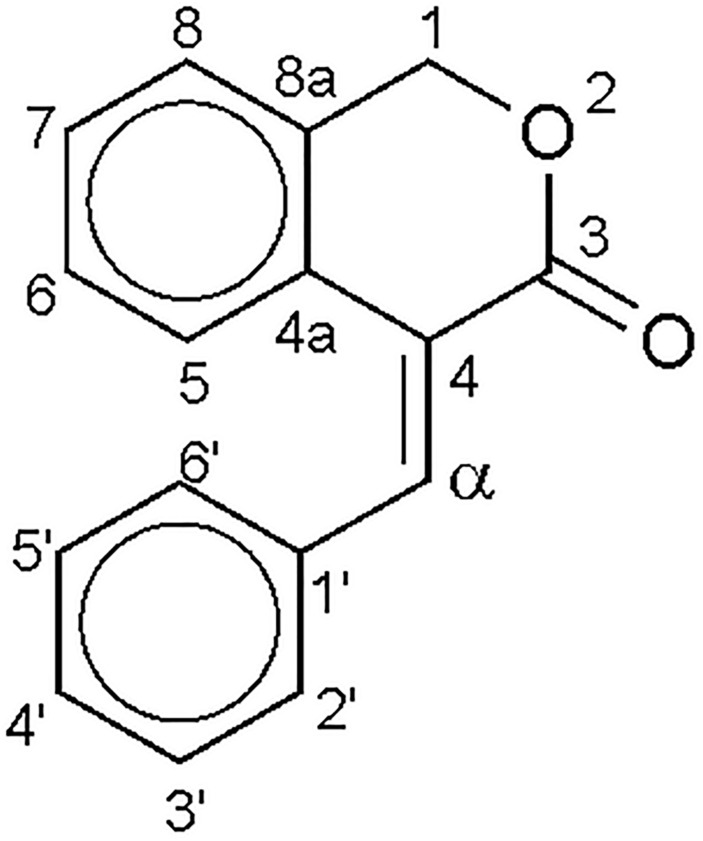
Numbering of the heavy atoms for the NMR assignments.

The main problem of the structure verification is the distinction between the *E-Z*-stereoisomers. The ^1^H- and ^13^C-APT NMR measurements of the substances achieved spectra in good accordance with the expected structures. As the stereochemistry of the double bond required further corroboration proven to be necessary earlier (Lóránd et al., 2002), with the help of the 2D HSQMBC technique (Williamson et al., 2000), the αH-C3 heteronuclear coupling constants ^3^J (see Table 3 and Figure 3) were determined. The *E*- and *Z*-possibilities of the same molecular framework had significantly and characteristically different proton-carbon coupling constants (see Table 3) allowing the confirmation of the expected geometries.

**TABLE 3:** The heteronuclear coupling constants of some compounds (alkenyl-H and carbonyl C).

**Compound**	**αH-C3 coupling constants (Hz)**	
	***E*-isomer**	***Z*-isomer**
**17E- 17Z**	7.1	12.8
**24E-24Z**	7.3	13.1
**27E-27Z**	7.4	12.9
**28E-28Z**	7.4	13.0

### Biological Part

#### Antifungal Assays and Structure-Activity Relationships

To study the structure-antifungal activity in the two molecular library of isochromanones (**1*E*-16*E*** and **17*E*-30*E***) different type of structures were studied. The effect of the structural changes on the antifungal effect was investigated. Thus the aromatic groups were either homo- or heteroaromatic, the substituents of the aromatic ring were selected as both electron withdrawing and electron donating ones. As for the influence of the stereochemical factor, the tested compounds are of *E*- or Z-configuration around the exocyclic double bond.

A series of yeasts was chosen for the antifungal screening as wild-type and mutants resistant to the specific fungal cell wall GS inhibitors papulacandins and echinocandins (Cortés et al., 2019): fission yeast *S. pombe* wild-type (972 h^–^) and *pbr1-8* and *pbr1-6* resistant mutants, budding yeast *S. cerevisiae* wild-type and *pbr1-1* resistant mutant, and the pathogenic dimorphic fungus *C. albicans* wild-type (ATCC 26555, SC5314 and ATCC 90028) (Castro et al., 1995; Martins et al., 2011). Four types of complete media were used for the fungal culture in the screening: standard YEPD, YES, YEPD+S (1.2 M sorbitol) and YES+S media were applied for the vegetative growth of yeast cells. Sorbitol is an osmotic stabilizer, and YEPD+S or YES+S media were used for the analysis of osmotic protection, either in cell wall deficient mutants or against the lethal antifungal effects (Ribas et al., 1991; Castro et al., 1995; Frost et al., 1995; Cortés et al., 2012; Muñoz et al., 2013).

The screening of the first generation of isochromanones was carried out in YEPD and YEPD+S media (**1*E*-16*E***). The fungal strains analyzed are: *C. albicans* wild-type ATCC 26555, SC5314 and ATCC 90028, *S. cerevisiae* wild-type CVX12-3A and *pbr1-1* mutant, and *S. pombe* wild-type (972h^–^), *pbr1-8* mutant and *pbr1-6* mutant. The results are summarized in Table 4. The bold numbers in Tables 4–6 indicate the more efficient compounds. In general, under these conditions the tested compounds (**1*E*-16*E***) showed a high activity. The weak or moderate efficacy against the *C. albicans* wild-type strains was observed by some substances (**3*E***, **4*Z***, **5*Z***, **10E**, **15,** and **16*E***). The efficiency toward the *S. pombe pbr1-6* mutant strain was the highest inhibitory activity, except **7*E*, 11*E*, 12** and **14*E***. The highest activity was observed at the **1*E***- phenyl derivative, **3*E*** –pyridyl derivative, **4*Z***- methylpyrrolyl compound, **5*Z***- pyrrolyl derivative, **8*Z***-2-chloro derivative. As regards the other *S. pombe pbr1-8* mutant strain, the antifungal activity of the tested compounds was slightly lower, but the substances with the best activity are partly identical to the best compounds mentioned with *pbr1-6*, (i.e., **1*E*,** **3*E***, **4*Z***, **5*Z)***.

**TABLE 4:** Antifungal activity of the first generation of isochromanones (**1–16**).

	**MIC (μg/ml; YEPD) Isolates**															
	***S. p.*^a^**		***S. p.*^b^**		***S. p.*^c^**		***S. c.*^d^**		***S. c.*^e^**		***C. a.*^f^**		***C. a.*^g^**		***C. a.*^h^**	
	**YEPD**	**YEPD + S**	**YEPD**	**YEPD + S**	**YEPD**	**YEPD + S**	**YEPD**	**YEPD + S**	**YEPD**	**YEPD + S**	**YEPD**	**YEPD + S**	**YEPD**	**YEPD + S**	**YEPD**	**YEPD + S**
**1*E***	**25**	200	**12.5**	**50**	**3.125**	**25**	100	100	**25**	**50**	200	**50**	200	>200	200	200
**1*Z***	**50**	100	**25**	100	**12.5**	**25**	100	200	200	100	>200	**50**	>200	>200	200	200
**2*E***	**50**	100	**50**	**50**	**25**	**50**	100	**25**	100	200	200	**50**	200	200	200	>200
**3*E***	**25**	100	**12.5**	**50**	**3.125**	**50**	**50**	**50**	**50**	100	100	**50**	100	100	**50**	**50**
**4*E***	**50**	100	**25**	**25**	**6.25**	**25**	100	**25**	**50**	200	200	**25**	200	200	200	200
**4*Z***	**25**	**25**	**6.25**	**12.5**	**3.125**	**12.5**	**12.5**	100	**25**	200	**25**	**50**	**50**	100	**12.5**	>200
**5*Z***	**25**	**50**	**6.25**	**25**	**3.125**	**6.25**	100	100	**50**	200	100	**12.5**	100	200	200	200
**6*Z***	**50**	100	**50**	**25**	**12.5**	**25**	100	200	100	200	200	**50**	200	>200	>200	>200
**7*E***	100	**50**	100	100	**50**	**50**	100	200	**50**	200	>200	200	200	>200	>200	200
**8*E***	**50**	100	**12.5**	**25**	**12.5**	**6.25**	100	200	**50**	200	>200	200	>200	200	200	>200
**8*Z***	**25**	**25**	**12.5**	**6.25**	**3.125**	**6.25**	100	200	**50**	100	200	**50**	>200	100	>200	100
**9*E***	**50**	100	**25**	**25**	**6.25**	**6.25**	100	100	**25**	200	100	100	100	100	100	200
**10*E***	100	**12.5**	**12.5**	**6.25**	**6.25**	**6.25**	100	200	**50**	200	>200	**50**	>200	100	>200	200
**11*E***	100	**6.25**	200	**25**	100	**12.5**	200	**50**	**50**	100	>200	**25**	>200	**25**	>200	**25**
**11*Z***	100	**50**	**50**	**50**	**25**	**25**	100	100	100	200	>200	**50**	>200	200	>200	100
**12**	200	**50**	200	**12.5**	**50**	**6.25**	100	**50**	100	**50**	>200	100	>200	200	>200	100
**13*E***	**25**	100	**25**	**12.5**	**12.5**	**12.5**	**50**	100	**50**	200	200	100	200	**50**	200	100
**14*E***	**50**	**12.5**	100	200	**25**	**25**	**25**	200	**25**	100	200	200	200	200	>200	>200
**15**	100	200	**50**	100	**12.5**	**50**	**50**	100	**25**	100	>100	**25**	>100	>200	>100	>200
**16*E***	100	200	100	100	**6.25**	**50**	200	200	100	200	200	200	200	>200	**50**	>200
**AMB**	<0.1	<0.1	0.1	0.1	<0.1	<0.1	0.2	0.2	0.2	0.2	0.4	0.4	0.8	0.8	0.4	0.4
**CSP**	5	Nd	50	Nd	50	nd	1	nd	100	Nd	50	nd	50	nd	50	Nd

*^*a*^*S. pombe* wild-type (972 h^–^), ^*b*^*S. pombe pbr 1-8*, ^*c*^*S. pombe pbr 1-6*, ^*d*^*S. cerevisiae* wild-type CVX 12-3A, ^*e*^*S. cerevisiae pbr 1-1*, ^*f*^*C. albicans* (ATCC 90028), ^*g*^*C. albicans* (ATCC 26555), ^*h*^*C. albicans* (SC5314). **AMB**, amphotericin B; **CSP**, caspofungin; **MIC**, minimal inhibitory concentration; nd, not determined.*

**TABLE 5:** MIC values of the first generation of isochromanones (**1*E*–16*E***) against different yeast strains.

	**MIC (μg/ml) isolates**											
	***S. p.*^a^**		***S. p.*^b^**		***S. p.*^c^**		***S. c.*^d^**		***S. c.*^e^**		***C. a.*^f^**	
	**YES**	**YES + S**	**YES**	**YES + S**	**YES**	**YES + S**	**YES**	**YES + S**	**YES**	**YES + S**	**YES**	**YES + S**
**1*E***	> 200	> 200	> 200	> 200	**20**	**50**	> 200	> 200	100	200	> 200	> 200
**1*Z***	> 200	> 200	> 200	> 200	**10**	**20**	> 200	> 200	> 200	> 200	> 200	> 200
**2*E***	> 200	> 200	> 200	> 200	200	200	> 200	> 200	> 200	> 200	> 200	> 200
**3*E***	200	> 200	200	> 200	**50**	100	200	> 200	200	> 200	> 200	> 200
**4*E***	> 200	> 200	> 200	> 200	200	200	> 200	> 200	> 200	> 200	> 200	> 200
**4*Z***	> 200	> 200	> 200	> 200	**50**	100	> 200	> 200	> 200	> 200	> 200	> 200
**5*Z***	> 200	> 200	> 200	> 200	**20**	**50**	> 200	> 200	> 200	> 200	> 200	> 200
**6*Z***	> 200	> 200	> 200	> 200	> 200	> 200	> 200	> 200	> 200	> 200	> 200	> 200
**7*E***	> 200	> 200	> 200	> 200	> 200	> 200	> 200	> 200	> 200	> 200	> 200	> 200
**8*E***	> 200	> 200	> 200	> 200	100	200	> 200	> 200	> 200	> 200	> 200	> 200
**8*Z***	> 200	> 200	> 200	> 200	100	200	> 200	> 200	> 200	> 200	> 200	> 200
**9*E***	> 200	> 200	> 200	> 200	> 200	> 200	> 200	> 200	> 200	> 200	> 200	> 200
**10*E***	> 200	> 200	> 200	> 200	> 200	> 200	> 200	> 200	> 200	> 200	> 200	> 200
**11*E***	> 200	> 200	> 200	> 200	> 200	> 200	> 200	> 200	> 200	> 200	> 200	> 200
**11*Z***	> 200	> 200	> 200	> 200	> 200	> 200	> 200	> 200	100	200	> 200	> 200
**12**	> 200	> 200	> 200	> 200	> 200	> 200	> 200	> 200	> 200	> 200	> 200	> 200
**13*E***	200	> 200	200	> 200	**50**	100	200	> 200	**50**	200	200	> 200
**14*E***	> 200	> 200	> 200	> 200	> 200	> 200	> 200	> 200	> 200	> 200	> 200	> 200
**15**	> 200	> 200	> 200	> 200	> 200	> 200	> 200	> 200	> 200	> 200	> 200	> 200
**16*E***	> 200	> 200	> 200	> 200	200	> 200	> 200	> 200	> 200	> 200	> 200	> 200
**AMB**	<0.1	<0.1	0.1	0.1	<0.1	<0.1	0.2	0.2	0.2	0.2	0.8	0.8
**CSP**	10	nd	50	Nd	50	nd	0.5	Nd	100	Nd	50	Nd

*^*a*^*S. pombe* wild-type (972 h^–^), ^*b*^*S. pombe pbr 1-8*, ^*c*^*S. pombe pbr 1-6*, ^*d*^*S. cerevisiae* wild-type (CVX12-3A), ^*e*^*S. cerevisiae pbr 1-1*, ^*f*^*C. albicans* wild-type (ATCC 26555). **AMB**, amphotericin B; **CSP**, caspofungin; **MIC**, minimal inhibitory concentration, assays in YES and YES+S medium; nd, not determined.*

**TABLE 6:** MIC values of the second generation of isochromanones (**17E-30E**) against different yeast strains.

	**MIC (μg/ml) isolates**													
	***S. p.*^a^**		***S. p.*^b^**		***S. p.*^c^**		***S. c.*^d^**		***S. c.*^e^**		***C. a.*^f^**		***C. a.*^g^**	
	**YES**	**YES + S**	**YES**	**YES + S**	**YES**	**YES + S**	**YES**	**YES + S**	**YES**	**YES + S**	**YES**	**YES + S**	**YES**	**YES + S**
**17*E***	> 200	> 200	200	> 200	**50**	100	200	> 200	> 200	> 200	> 200	> 200	> 200	> 200
**17*Z***	> 200	> 200	**50**	200	**5**	**50**	> 200	> 200	> 200	> 200	> 200	> 200	> 200	> 200
**18*E***	> 200	> 200	> 200	> 200	> 200	> 200	> 200	> 200	> 200	> 200	> 200	> 200	> 200	> 200
**19*E***	> 200	> 200	> 200	> 200	**5**	**50**	> 200	> 200	> 200	> 200	> 200	> 200	> 200	> 200
**20*E***	> 200	> 200	**20**	> 200	**20**	100	> 200	> 200	> 200	> 200	> 200	> 200	> 200	> 200
**21*E***	> 200	> 200	200	200	**10**	200	> 200	> 200	> 200	> 200	> 200	> 200	> 200	> 200
**22*E***	> 200	> 200	**10**	100	**10**	100	> 200	> 200	> 200	> 200	> 200	> 200	> 200	> 200
**23*E***	> 200	> 200	> 200	> 200	**5**	> 200	> 200	> 200	> 200	> 200	> 200	> 200	> 200	> 200
**24*E***	> 200	> 200	> 200	> 200	> 200	> 200	> 200	> 200	> 200	> 200	> 200	> 200	> 200	> 200
**24*Z***	> 200	> 200	> 200	> 200	> 200	> 200	> 200	> 200	> 200	> 200	> 200	> 200	> 200	> 200
**25*E***	> 200	> 200	200	> 200	> 200	> 200	> 200	> 200	> 200	> 200	> 200	> 200	> 200	> 200
**26*E***	> 200	> 200	100	> 200	**20**	**50**	> 200	> 200	> 200	> 200	> 200	> 200	> 200	> 200
**27*E***	> 200	> 200	> 200	> 200	150	> 200	> 200	> 200	> 200	> 200	> 200	> 200	> 200	> 200
**28*E***	> 200	> 200	> 200	> 200	**5**	200	> 200	> 200	> 200	> 200	> 200	> 200	> 200	> 200
**28*Z***	> 200	> 200	> 200	> 200	**5**	100	> 200	> 200	> 200	> 200	> 200	> 200	> 200	> 200
**29*E***	> 200	> 200	> 200	> 200	> 200	> 200	> 200	> 200	> 200	> 200	> 200	> 200	> 200	> 200
**30*E***	150	> 200	150	200	**50**	100	150	200	150	200	> 200	> 200	> 200	> 200
**AMB**	<0.1	<0.1	0.1	0.1	<0.1	<0.1	0.2	0.2	0.2	0.2	0.8	0.8	0.4	0.4
**CSP**	10	Nd	50	nd	50	nd	0.5	nd	100	nd	50	nd	50	nd

*^*a*^*S. pombe* wild-type (972 h^–^), ^*b*^*S. pombe pbr 1-8*, ^*c*^*S. pombe pbr 1-6*, ^*d*^*S. cerevisiae* wild-type (CVX12-3A), ^*e*^*S. cerevisiae pbr 1-1*, ^*f*^*C. albicans* (ATCC 26555), ^*g*^*C. albicans* (SC5314). **AMB**, amphotericin B; **CSP**, caspofungin; **MIC**, minimal inhibitory concentration, assays in YES and YES+S medium. nd, not determined.*

This family of isochromanones showed weak or moderate antifungal activity toward the *C. albicans* wild-type strains (**3*E***, **4*Z***, **5*Z***, **9*E*** and **16*E***), and **4*Z***- methylpyrrolyl compound proved to be the most active one

Next, the first generation of isochromanones (**1*E*-16*E***) was also screened in YES medium (Table 5) and exerted a weaker antifungal potency than in YEPD medium and also than the second family of isochromanones in YES medium (**17*E*-30*E***) (Table 6). The strains are partly different from the previously mentioned ones (Tables 5, 6) analyzing two wild type *C. albicans* strains because they behaved similarly in YEPD. It is remarkable that there was no effect against either the *C. albicans*, the *S. cerevisiae*, or the *S. pombe* wild-type strains, except with **30*E***. Interestingly, the *S. cerevisiae* and more specially the *S. pombe pbr1-6* and *pbr1-8* mutants resistant to specific fungal cell wall inhibitors showed different degrees of sensitivity. The results of these antifungal assays in YES medium are displayed in Tables 5, 6.

The *S. pombe pbr1-6* mutant strain was the more vulnerable one, as several isochromanones of the first generation exerted a medium or good antifungal effect (e.g., **1*E*-1*Z***- phenyl derivatives, **3*E*** –pyridyl derivative, **4*Z*** –methylpyrrolyl, **5*Z*** - pyrrolyl compound and **13E** furyl derivative). The *S. cerevisiae* strains were less sensitive toward the isochromanones, from the first generation of the isochromanones almost only one substance, the **13*E*** furyl derivative - the most efficient antifungal from this series- proved to be efficient and **1*E*** and **11*E*** to be slightly efficient against the *S. cerevisiae pbr1-1* mutant strains.

We wanted to see the bioactivity of the second family of isochromanones to see if they had a better antifungal activity in YES with respect to WT and pbr mutants. This family of compounds (**17*E*-30*E***), generally showed higher antifungal activity than the first generation (Tables 5, 6). With the exception of **30*E***- a vanillin derivative – all of the compounds were inefficient against the wild-type strains of *S. pombe*, *S. cerevisiae* and *C. albicans*. Several substances proved to be very active specially against the *S. pombe pbr1-6* mutant strain, some compounds with electron withdrawing groups as **17*Z* -**4-nitrophenyl derivative, **23*E* -**4-chlorophenyl derivative, **28*E*-28*Z*** -3-chlorophenyl derivatives, but some substances with electron donating groups also exerted an excellent antifungal potency as **19*E*** -4-methylphenyl derivative, **21*E*** 4-methoxyphenyl derivative. Some compounds showed a medium antifungal potency (as **20*E***, **26*E***). As regards the antifungal activity of the *E*-*Z* stereoisomers, there was a difference in the potency of **17*E*** and **17*Z***, the latter was ten times more efficient.

Most of the previously mentioned compounds showed antifungal activity also against the *S. pombe pbr1-8* mutant strain, although in some cases it was weaker being the most efficient: **17*Z* -**4-nitrophenyl derivative, **20*E*** -2-methylphenyl derivative, **22*E-*** 3-methoxyphenyl derivative. Out of the second generation of isochromanones the **30*E –*** vanillin derivative - had the broadest spectrum showing antifungal activity against all of the strains except the *C. albicans* wild-type strains.

The type of medium i. e. composition, incubation times and temperature can influence the growth and detectability of fungi from the clinical samples and the MIC values of the tested strains (Als et al., 2010; Cruz et al., 2013; Balouiri et al., 2016). The Sabouraud and YEPD media are effective and most useful as media for subculture (Washington et al., 2006). Modified RPMI medium is proposed as a standard medium for determination of MIC values of fungi (Washington et al., 2006; Espinel-Ingroff and Pfaller, 2007; Clinical and Laboratory Standard Institute, 2008). In our experiments we used the standard YES and YEPD media and they were optimal for our experimental strains and assays.

Summarizing the results of the antifungal screenings, the most efficient compounds with activity and the lowest MIC values against several strains are the following compounds ordered by their antifungal activity: **1*E***- phenyl derivative, **3*E*** –pyridyl derivative, **4*Z***- methylpyrrolyl compound, **5*Z***- pyrrolyl derivative, **8*Z***-2-chloro derivative, **9*E*** – 2,3-dihydroxy derivative and the **15** coumarine from the first family, from the second family the **17*Z* -**4-nitrophenyl, **19*E*** -4-methylphenyl, **23*E* -**4-chlorophenyl and **28*E*-28*Z*** -3-chlorophenyl derivatives.

#### Mode of Action

In order to explore the mechanism of action of the tested compounds, the osmotic protection against the antifungal effect was analyzed. The inhibitory effect of the compounds in YEPD and YES media were compared with their inhibitory effect in YEPD or YES medium containing 1.2 M sorbitol (YEPD+S or YES+S). These media have been used to detect osmotically fragile mutants that can be rescued in the presence of sorbitol. These mutants present defects in their cell wall synthesis and composition (Ribas et al., 1991; Cortés et al., 2012; Muñoz et al., 2013). Similarly, the osmotic protection can partially protect the cells against the specific cell wall synthesis antifungals (Frost et al., 1995). Therefore, we tested in YEPD+S and YES+S the inhibitory effect of the compounds that showed some *in vivo* inhibition in YES medium (Tables 4–6). In all of the cases sorbitol protected the cells increasing the *in vivo* MIC of the tested compound (Tables 4–6), suggesting that the mode of action of the compounds could be inhibiting the synthesis of essential cell wall polymers, as it has been described for the specific GS inhibitors papulacandins and echinocandins (Martins et al., 2011; Brown et al., 2012; Kathiravan et al., 2012; Cortés et al., 2019). To explore this possibility of the mode of action of the tested drugs, their putative inhibitory capacity of the enzymatic GS activity was also examined *in vitro* (Tables 7, 8). According to these tests, the first family of isochromanones acted as poor inhibitor of this enzyme, compounds **1*E*** – **1*Z*** showed weak inhibitory effect against the *in vitro* GS of *S. pombe pbr1-6* mutant strain (Table 7). Other compounds that had no *in vivo* effect showed some weak *in vitro* inhibitory effect detected as IC30 (30% of the maximal inhibitory concentration) but not as IC50 (half-maximal inhibitory concentration), indicating that some *in vitro* inhibition was present but it never or hardly reached the 50% of inhibition. Interestingly, the *in vitro* inhibition was higher in the GS enzyme of *pbr1-6* mutant, as observed *in vivo* in living cells. All these data are in accordance with their weak antifungal effect.

**TABLE 7:** Inhibitory capacity of the first generation of isochromanones of the β(1-3)glucan synthase (*in vitro* assay) expressed as IC_50_ and IC_30_ values.

	**IC_50_ and IC_30_ antifungals (μg/ml)**															
**Isolates**	**1*E***		**1*Z***		**2*E***		**3*E***		**4*E***		**4*Z***		**5*Z***		**6*Z***	
	**IC 50**	**IC 30**	**IC 50**	**IC 30**	**IC 50**	**IC 30**	**IC 50**	**IC 30**	**IC 50**	**IC 30**	**IC 50**	**IC 30**	**IC 50**	**IC 30**	**IC 50**	**IC 30**
*S. pombe* (WT)	250	125	250	25	500	250	250	25	250	25	500	50	500	125	> 500	250
*S. pombe pbr 1-6*	125	50	125	25	> 500	250	250	125	250	50	> 500	25	> 500	125	> 500	250

**Isolates**	**7*E***		**8*E***		**8*Z***		**9*E***		**10*E***		**11*E***		**11*Z***		**12**	
	**IC 50**	**IC 30**	**IC 50**	**IC 30**	**IC 50**	**IC 30**	**IC 50**	**IC 30**	**IC 50**	**IC 30**	**IC 50**	**IC 30**	**IC 50**	**IC 30**	**IC 50**	**IC 30**

*S. pombe* (WT)	> 500	500	500	125	> 500	250	500	125	> 500	500	250	25	> 500	500	> 500	500
*S. pombe pbr 1-6*	> 500	250	250	25	500	250	500	125	500	250	250	25	500	250	250	125

**Isolates**	**13*E***		**14*E***		**15*E***		**16*E***		**CSP**							
	**IC 50**	**IC 30**	**IC 50**	**IC 30**	**IC 50**	**IC 30**	**IC 50**	**IC 30**	**IC 50**	**IC 30**						

*S. pombe* (WT)	> 500	125	500	125	500	50	250	25	0.3	0.08						
*S. pombe pbr 1-6*	250	125	250	25	250	50	250	25	150	1						

*Isolates: *Schizosaccharomyces pombe* wild-type (WT, 972 h^–^) and *pbr1-6* papulacandin resistant. **CSP**, Caspofungin.*

**TABLE 8:** Inhibitory capacity of the second generation of isochromanones of the β(1-3)glucan synthase (*in vitro* assay) expressed as IC_50_ and IC_30_ values.

	**IC_50_ and IC_30_ of antifungals (μg/ml)**															
**Isolates**	**17*E***		**17*Z***		**18*E***		**19*E***		**20*E***		**21*E***		**22*E***		**23*E***	
	**IC 50**	**IC 30**	**IC 50**	**IC 30**	**IC 50**	**IC 30**	**IC 50**	**IC 30**	**IC 50**	**IC 30**						
*S. pombe* (WT)	> 500	100	> 500	> 500	> 500	> 500	> 500	> 500	> 500	250	> 500	> 500	> 500	> 500	> 500	225
*S. pombe pbr 1-8*	250	25	> 500	> 500	> 500	> 500	> 500	500	500	500	> 500	250	500	100	250	125
*S. pombe pbr 1-6*	25	<0.2	50	5	50	<02	50	12.5	125	12.5	> 500	250	50	10	125	12.5

**Isolates**	**24*E***		**24*Z***		**25*E***		**26*E***		**27*E***		**28*E***		**28*Z***		**29*E***	
	**IC 50**	**IC 30**	**IC 50**	**IC 30**	**IC 50**	**IC 30**	**IC 50**	**IC 30**	**IC 50**	**IC 30**						

*S. pombe* (WT)	> 500	> 500	> 500	> 500	> 500	100	500	125	> 500	> 500	125	37.5	500	150	500	250
*S. pombe pbr 1-8*	> 500	> 500	> 500	> 500	175	50	150	12.5	500	50	250	175	250	50	> 500	> 500
*S. pombe pbr 1-6*	> 500	> 500	> 500	> 500	> 500	> 500	125	25	125	50	125	50	250	50	> 500	250

**Isolates**	**30*E***		**CSP**													
	**IC 50**	**IC 30**	**IC 50**	**IC 30**												

*S. pombe* (WT)	125	62.5	0.3	0.08												
*S. pombe pbr 1-8*	125	12.5	250	175												
*S. pombe pbr 1-6*	50	5	150	1												

*Isolates: *Schizosaccharomyces pombe* wild-type (WT, 972 h^–^), and *pbr1-8* and *pbr1-6* papulacandin resistant. **CSP**, Caspofungin.*

Regarding the second generation of isochromanones, they produced a much higher inhibitory effect against the GS of *S. pombe pbr1-6* mutant strain (as **17*E*–17*Z* –** 4-nitrophenyl, **18*E*** – 2,4,6-trimethylphenyl, **19*E*** – 4-methylphenyl, **22*E* –** 3-methoxyphenyl and the **30*E*** - vanillin derivative, Table 8). The inhibition of *pbr1-6* GS was even stronger considering the IC30 value compared to that of wild-type GS. Concerning the influence of the aromatic moieties there are both electron withdrawing and electron donating ones among these substituents. The inhibition of these compounds on the other *S. pombe pbr1-8* mutant strain was slight (**17*E*, 23*E*, 25*E*, 26*E*, 28*E*, 28*Z*,** and **30*E***). As before, the effect was more pronounced considering the IC30. Finally, the wild-type GS also showed a noticeable sensitivity to drug **30*E***. These promising results open new possibilities of antifungal strategies that will require, however, to be explored in deep with echinocandin resistant mutants of pathogenic fungi in future studies.

## Implications

A synthetic route yielding differently substituted *E*- and *Z*-arylidene-3-isochromanones was applied. These condensations generally afforded the *E*-isomer, but the aromatic substituent has an impact on the stereocomposition of the reaction mixture. The NMR measurements (the αH-C3 heteronuclear coupling constants) afforded unequivocal proof for the steric assignments.

The antifungal effect of the two generation of isochromanones against wild-type and mutant yeast strains showed big difference in the antifungal efficacy. The first generation of isochromanones were less efficient toward the wild-type and mutant strains. Several compounds of the second generation of isochromanones as **17*E*-*Z*, 19*E*, 21*E*, 23*E*, 26*E*, 28*E*-*Z*** of different substitution pattern showed good or medium activity mainly against the *S. pombe pbr1-6* and some against the *pbr1-8* mutant strain. A similar effect with some compounds **1*E*, 11*Z*, 13*E*** and **30*E*** was also observed with *S. cerevisiae* wild-type and *pbr1-1* mutant. The *C. albicans* wild-type strains showed sensitivity toward the isochromanones (**3*E*, 4*Z*, 5*Z* 9*E*** and **16*E***) in YEPD medium but were not sensitive in YES medium. The stereochemical factors – *E-Z* isomerism or substitution pattern of the aromatic ring had an impact on the bioactivity. The first generation of isochromanones was screened using different culture media and the MIC values obtained were different. These experiments demonstrated the effect of the medium on antifungal activity. At last the mode of action was also investigated. According to the results our isochromanones proved to be weak inhibitor of the wild-type GS enzyme but potent inhibitors of the echinocandin resistant GS enzymes.

Susceptibility to antimicrobial drugs depends on the isolated strains and the technique used for determination of susceptibility. The first and very sensitive group of our tested yeast strains was isolated in Kuwait more than 20 years ago (Lóránd et al., 1998). There are different endemic strains in these countries, and there are different inhibition protocols that could show different sensitivities. The type of medium (YES, YEPD, or Casamino medium in the first test), time and temperature of strains incubation, test methods (micro and macro tube dilution, micro plate dilution or E – test) used for determination of the antifungal sensitivity can influence the MIC values. We have to take into consideration these facts when trying to compare MIC values of strains isolated in Kuwait more than 20 years ago with standard laboratory strains either wild-type or isolated mutants resistant to GS antifungals.

Independently of the sensitivity test used, we demonstrated that the isochromanones inhibit the synthesis of cell wall β(1,3)-glucan and that this inhibition is specially enhanced in mutants resistant to the GS inhibitors papulacandins and echinocandins, indicating that isochromanones present a different mechanism of action than the described cell wall β(1,3)-glucan inhibitors papulacandins, acidic terpenoids and echinocandins (Martins et al., 2011; Cortés et al., 2019). This higher sensitivity of the mutants resistant to echinocandins may provide new insights into new strategies of combined antifungal therapy using echinocandins and isochromanones, directed against the spontaneous emergence of mutants resistant to echinocandins.

## Author Contributions

JR, MC, and TL designed the experiments and revised the manuscript. TL performed the synthesis of the test compounds and the FT-IR examinations, RB, MC and BK performed the biological experiments and MC analyzed the biological data. AA and GG-F conducted the structure verifications by NMR methods. JR, TL, RB, GG-F, and AA wrote the manuscript.

## Conflict of Interest Statement

The authors declare that the research was conducted in the absence of any commercial or financial relationships that could be construed as a potential conflict of interest.
