# Supplementary material for: Novel Cell Wall Antifungals Reveal a Special Synergistic Activity in pbr1 Mutants Resistant to the Glucan Synthesis Antifungals Papulacandins and Echinocandins
Source: Front Microbiol. 2019 Jul 24;10:1692. doi: 10.3389/fmicb.2019.01692 (PMC6689975; doi:10.3389/fmicb.2019.01692)
Supplement: Supplementary file 1 [file Data_Sheet_1.pdf]

Supplementary Material

Novel cell wall antifungals reveal a special synergistic activity in mutants resistant to the glucan synthesis antifungals papulacandins and echinocandins

Rodrigo Berzaghi, Attila Agócs, Gergely Gulyás-Fekete, Béla Kocsis, Juan C. Ribas, Tamás Lóránd*

*** Correspondence:** Tamás Lóránd: tamas.lorand@aok.pte.hu

1 Supplementary Data

1.1 Supplementary Material- Experimental part detailed

General information

All of the reagents were purchased from Sigma-Aldrich Company and were used without further purifications. The purification of the novel compounds was performed with column chromatography on Sigma-Aldrich silica gel (pore size: 60 Å, particle size 230-400 mesh). The analytical thin-layer chromatography was performed on Merck silica gel plates (60 F254) and the eluents used are described in the next chapter. NMR spectra were recorded on a Bruker Avance III Ascend 500 spectrometer (500/125 MHz for 1H/13C); chemical shifts are referenced to residual solvent signals. Measurements were performed at a probe temperature of 298 K in solution with an appropriate solvent. The FT IR spectra were run on an Impact 400 (Nicolet) FT IR spectrophotometer in KBr pellets using a KBr pellet as the background reference spectrum. Infrared spectra were obtained between 400 and 4000 cm-1 with a spectral resolution of 4 cm-1 Melting points were measured with a Boethius hot plate apparatus and are uncorrected.

Synthesis

General procedure for the synthesis of 4-arylmethylene-3-isochromanones (17-30) given as an example of compound **30*E***

Prepared from a mixture of 3-isochromanone (0.50 g; 3.38 mmol) and vanillin (0.51 g, 3.38 mmol) with the above method. The reaction mixture was purified via column chromatography (silica gel, dichloromethane/methanol=10:0.05) to give **30*E*** as a faint yellow crystalline solid from methanol (0.60g, 63%), Rf=0.24 (silica gel, dichloromethane/methanol=10:0.1), m.p. 140-141 °C. IR νmax (cm-1) (KBr) 3346 (st, OH), 1696 (st C=O). 1H NMR (500 MHz, dmso-d6) δ (ppm) 3.59 (s, 3H), 5.34 (s, 2H), 6.75 (d, J = 8.2 Hz, 1H), 7.05 (dd, J = 8.4, 1.7 Hz, 1H), 7.16 (d, J = 1.9 Hz, 1H), 7.27 (dt, J = 7.7,

1.3 Hz, 1H), 7.33 (dt, J = 7.5, 1.2 Hz, 1H), 7.42 (d, J = 7.5 Hz, 1H), 7.52 (d, J = 7.4 Hz, 1H), 7.60 (s, 1H). 13C NMR (125 MHz, dmso-d6) δ (ppm) 55.3, 68.5, 113.1, 115.5, 121.8, 124.1, 124.6, 125.7, 126.5, 127.5, 128.1, 130.5, 133.0, 138.1, 147.2, 148.8, 168.2. 3J(Hα-C3) = 7.9 Hz. Anal Calcd for C17H14O4: C, 72.33; H, 5.00; Found: C, 72.41; H, 5.12.

Spectral data of other compounds

***E*-4-[(4'-nitrophenyl)methylene]-3-isochromanone (17*E*)**

1H NMR (500 MHz, dmso-d6) δ (ppm) 5.47 (s, 2H), 7.13 (d, J = 7.6 Hz, 1H), 7.20 (t, J = 7.2 Hz, 1H), 7.38 (dt, J = 7.6, 0.7 Hz, 1H), 7.47 (d, J = 7.6 Hz, 1H), 7.72 (d, J = 8.7 Hz, 2H), 7.79 (s, 1H), 8.21 (d, J = 8.7 Hz, 2H).13C NMR. (125 MHz, dmso-d6) δ (ppm) 68.8, 123.7, 125.8, 127.0, 128.1, 128.2, 129.0, 129.1, 130.4, 133.5, 135.0, 141.3, 147.2, 167.1, 3J(Hα-C3) = 7.1 Hz.

***Z*-4-[(4'-nitrophenyl)methylene]-3-isochromanone (17*Z*)**

1H-NMR (500 MHz, dmso-d6) δ (ppm) 5.38 (s, 2H), 7.39 (m, 2H), 7.49 (t, J = 7.5 Hz, 1H), 7.54 (d, J = 7.9 Hz, 1H), 7.60 (t, J = 7.7 Hz, 1H), 7.72 (m, 2H), 7.88 (s, 1H), 8.16 (d, J = 7.9 Hz, 1H). 13C NMR (125 MHz, dmso-d6) δ (ppm) 69.5, 124.9, 125.5, 126.0, 129.5, 129.9, 131.8, 131.9, 132.5, 133.0, 134.6, 137.4, 147.3, 165.7. 3J(Hα-C3) = 12.8 Hz.

***E*- 4-[(2',3',4'-Trimethylphenyl)methylene]-3-isochromanone (18*E*)**

1H NMR (500 MHz, CDCl3) δ (ppm) 2.07 (s, 6H), 2.30 (s, 3H), 5.34 (s, 2H), 6.80 (d, J = 7.9 Hz, 1H), 6.87 (s, 2H), 7.02 (dt, J = 8.0, 2.0 Hz, 1H), 7.21 (m, 2H), 7.95 (s, 1H).13C NMR (125 MHz, CDCl3) δ (ppm) 19.9, 21.0, 69.0, 124.5, 125.9, 126.7, 128.1, 128.3, 128.7, 131.0, 131.1, 131.2, 135.1, 137.8, 139.1, 167.9, 3J(Hα-C3) = 7.2 Hz.

***E* -4-[(4'-Methylphenyl)methylene]-3-isochromanone (19*E*)**

1H NMR (500 MHz, CDCl3) δ (ppm) 2.36 (s, 3H), 5.30 (s, 2H), 7.11 (d, J = 8.0 Hz, 2H), 7.15 (dt, J = 8.0, 2.0 Hz, 1H), 7.22-7.30 (br m, 2H), 7.39 (d, J = 8.0 Hz, 2H), 7.45 (d, J = 7.8 Hz, 1H), 7.81 (s, 1H).13C NMR (125 MHz, CDCl3) δ (ppm) 21.5, 69.2, 124.1, 125.2, 127.3, 127.9, 128.3, 129.3, 129.4, 130.7, 131.4, 132.4, 139.0, 140.0, 168.8, 3J(Hα-C3) = 7.3 Hz.

***E* -4-[(2'-Methylphenyl)methylene]-3-isochromanone (20*E*)**

1H NMR (500 MHz, CDCl3) δ (ppm) 2.37 (s, 3H), 5.36 (s, 2H), 7.05-7.11 (br m, 3H), 7.21-7.30 (br m, 5H), 8.03 (s, 1H).13C NMR (125 MHz, CDCl3) δ (ppm) 19.9, 69.2, 124.9, 125.8, 125.9, 127.4, 128.0, 128.2, 128.3, 129.1, 130.5, 130.6, 132.0, 134.1, 137.0, 138.6, 168.2, 3J(Hα-C3) = 7.8 Hz.

***E* -4-[(4'-Methoxyphenyl)methylene]-3-isochromanone (21*E*)**

1H NMR (500 MHz, CDCl3) δ (ppm) 3.83 (s, 3H), 5.29 (s, 2H), 6.82 (d, J = 8.7 Hz, 2H), 7.17 (dt, J = 7.9, 2.3 Hz, 1H), 7.22-7.30 (br m, 2H), 7.47 (d, J = 8.7 Hz, 2H), 7.52 (d, J = 7.9 Hz, 1H), 7.76 (s, 1H).13C NMR (125 MHz, CDCl3) δ (ppm) 55.3, 69.2, 114.0, 122.9, 125.3, 126.6, 127.1, 127.9, 128.2, 130.9, 131.3, 132.5, 138.7, 160.8, 169.0, 3J(Hα-C3) = 7.4 Hz.

***E* -4-[(3'-Methoxyphenyl)methylene]-3-isochromanone (22*E*)**

1H NMR (500 MHz, CDCl3) δ (ppm) 3.71 (s, 3H), 5.32 (s, 2H), 6.87 (dd, J = 8.0, 2.3 Hz, 1H), 7.00 (s, 1H), 7.05 (d, J = 7.8 Hz, 1H), 7.16 (dt, J = 7.8, 1.7 Hz, 1H), 7.19-7.30 (br m, 3H), 7.42 (d, J = 8.0 Hz, 1H), 7.80 (s, 1H).13C NMR (125 MHz, CDCl3) δ (ppm) 55.2, 69.3, 114.2, 115.5, 121.9, 125.2, 125.3, 127.6, 127.9, 128.5, 129.7, 130.3, 132.5, 135.6, 138.6, 159.6, 168.6, 3J(Hα-C3) = 7.7 Hz.

***E* -4-[(4'-Chlorophenyl)methylene]-3-isochromanone (23*E*)**

1H NMR (500 MHz, CDCl3) δ (ppm) 5.32 (s, 2H), 7.16 (dt, J = 7.8, 1.6 Hz, 1H), 7.25-7.32 (br m, 4H), 7.36 (d, J = 7.8 Hz, 1H), 7.40 (d, J = 8.4 Hz, 2H), 7.75 (s, 1H). 13C NMR (125 MHz, CDCl3) δ (ppm) 69.3, 125.4, 125.6, 127.3, 128.1, 128.7, 128.9, 130.0, 130.6, 132.6, 132.7, 135.3, 137.2, 168.4. 3J(Hα-C3) = 7.5 Hz.

***E*-4-[(3'-nitrophenyl)methylene]-3-isochromanone (24*E*)**

1H NMR (500 MHz, CDCl3) δ (ppm) 5.46 (s, 2H), 7.19 (m, 2H), 7.37 (m, 1H), 7.47 (d, J = 7.6 Hz, 1H), 7.65 (t, J = 8.0 Hz, 1H), 7.78 (s, 1H), 7.90 (d, J = 7.7 Hz, 1H), 8.19 (dd, J = 8.2, 1.5 Hz, 1H), 8.32 (s, 1H).13C NMR (125 MHz, CDCl3) δ (ppm) 68.8, 123.5, 123.7, 125.8, 126.6, 127.5, 127.9, 128.9, 129.0, 130.1, 133.4, 134.8, 135.7, 135.9, 147.8, 167.2. 3J(Hα-C3) = 7.3 Hz.

***Z*-4-[(3'-nitrophenyl)methylene]-3-isochromanone (24*Z*)**

1H NMR (500 MHz, dmso-d6) δ (ppm) 5.47 (s, 2H), 7.43 (m, 2H), 7.50 (t, J = 7.3 Hz, 1H), 7.70 (m, 2H), 7.86 (d, J = 7.6 Hz, 1H), 8.10 (d, J = 7.7 Hz, 1H), 8.20 (m, 1H), 8.63 (s, 1H). 13C NMR (125 MHz, dmso-d6) δ (ppm) 68.6, 123.3, 124.0, 124.2, 124.7, 126.8, 128.6, 128.9, 129.3, 131.9, 133.2, 136.3, 136.5, 136.7, 147.4, 165.1, 3J(Hα-C3) = 13.1 Hz.

***E* -4-[(3',4',5'-Trimethoxyphenyl)methylene]-3-isochromanone (25*E*)**

1H NMR (500 MHz, CDCl3) δ (ppm) 3.70 (s, 6H), 3.88 (s, 3H), 5.31 (s, 2H), 6.74 (s, 2H), 7.20 (dt, J = 7.9, 2.0 Hz, 1H), 7.28 (m, 2H), 7.57 (d, J = 7.8 Hz, 1H), 7.72 (s, 1H). 13C NMR (125 MHz, CDCl3) δ (ppm) 56.0, 60.9, 69.2, 106.9, 124.2, 125.3, 127.5, 127.6, 128.5, 129.2, 130.4, 132.6, 138.5, 153.1, 168.8. 3J(Hα-C3) = 7.7 Hz.

***E* -4-[(3',4'-Dimethoxyphenyl)methylene]-3-isochromanone (26*E*)**

1H NMR (500 MHz, CDCl3) δ (ppm) 3.67 (s, 3H), 3.90 (s, 3H), 5.29 (s, 2H), 6.80 (d, J = 8.4 Hz, 1H), 7.06 (d, J = 1.9 Hz, 1H), 7.11 (dd, J = 8.8, 1.9 Hz, 1H), 7.19 (m, 1H), 7.27 (m, 2H), 7.56 (d, J = 7.8 Hz, 1H), 7.75 (s, 1H). 13C NMR (125 MHz, CDCl3) δ (ppm) 55.7, 55.9, 69.2, 110.9, 111.8, 122.9, 124.1, 125.3, 126.6, 127.3, 127.6, 128.2, 130.7, 132.5, 138.7, 148.6, 150.5, 169.0. 3J(Hα-C3) = 7.6 Hz.

***E* -4-[(3',4'-Methylenedioxyphenyl)methylene]-3-isochromanone (27*E*)**

1H NMR (500 MHz, dmso-d6) δ (ppm) 5.30 (s, 2H), 5.97 (s, 2H), 6.87 (d, J = 8.1 Hz, 1H), 6.95 (m, 1H), 7.04 (d, J = 8.4 Hz, 1H), 7.20 (t, J = 7.5 Hz, 1H), 7.31 (m, 2H), 7.39 (d, J = 7.3 Hz, 1H), 7.55 (s, 1H). 13C NMR (125 MHz, dmso-d6) δ (ppm) 69.6, 102.3, 1.09.0, 109.5, 124.2, 126.1, 126.6, 127.2, 128.3, 128.7, 129.4, 130.6, 133.7, 138.4, 148.0, 149.4, 169.5. 3J(Hα-C3) = 7.4 Hz.

***Z* -4-[(3',4'-Methylenedioxyphenyl)methylene]-3-isochromanone (27*Z*)**

1H NMR (125 MHz, dmso-d6) δ (ppm) 5.30 (s, 2H), 6.00 (s, 2H), 6.91 (d, J = 8.1 Hz, 1H), 7.21 (dd, J = 8.5, 1.5 Hz, 1H), 7.36 (m, 3H), 7.43 (m, 2H), 7.68 (d, J = 7.8 Hz, 1H). 13C NMR (125 MHz, dmso-d6) δ (ppm) 69.3, 102.1, 108.8, 110.0, 123.6, 124.1, 125.3, 127.3, 128.6, 128.9, 129.9, 132.1, 134.9, 140.2, 147.7, 149.0, 167.1, 3J(Hα-C3) = 12.9 Hz.

***E* -4-[(3'-Chlorophenyl)methylene]-3-isochromanone (28*E*)**

1H NMR (500 MHz, CDCl3) δ (ppm) 5.33 (s, 2H), 7.17 (dt, J = 7.7, 1.5 Hz, 1H), 7.25-7.37 (br m, 6H), 7.44 (m, 1H), 7.75 (s, 1H). 13C NMR (125 MHz, CDCl3) δ (ppm) 69.3, 125.3, 126.3, 127.3, 127.4, 128.1, 128.8, 128.9, 129.3, 129.7, 129.9, 132.5, 134.5, 136.1, 136.7, 168.1. 3J(Hα-C3) = 7.4 Hz.

***Z* -4-[(3'-Chlorophenyl)methylene]-3-isochromanone (28*Z*)**

1H NMR (500 MHz, CDCl3) δ (ppm) 5.33 (s, 2H), 7.20 (s, 1H), 7.27 (m, 1H), 7.34 (m, 2H), 7.39 (dt, J = 7.5, 1.1 Hz, 1H), 7.46 (t, J = 7.5 Hz, 1H), 7.61 (m, 2H), 7.71 (s, 1H). 13C NMR (125 MHz, CDCl3) δ (ppm) 69.3, 123.9, 124.4, 126.6, 128.3, 128.5, 129.3, 129.4, 129.5, 130.0, 131.6, 134.2, 134.5, 136.0, 138.1, 165.4. 3J(Hα-C3) = 13.0 Hz.

***E* -.4-[(4'-Hydroxyphenyl)methylene]-3-isochromanone (29*E*)**

1H NMR (500 MHz, dmso-d6) δ (ppm) 5.34 (s, 2H), 6.73 (d, J = 8.7 Hz, 2H), 7.23 (dt, J = 7.7, 1.2 Hz, 1H), 7.32 (dt, J = 7.5, 1.0 Hz, 1H), 7.42 (m, 4H), 7.60 (s, 1H).13C NMR (125 MHz, dmso-d6) δ (ppm) 68.4, 115.5, 121.9, 124.4, 125.7, 126.2, 127.7, 128.0, 130.5, 131.4, 132.9, 138.0, 159.1, 168.1.3J(Hα-C3) = 7.3 Hz.
